# Surveillance to Track Progress Toward Polio Eradication — Worldwide, 2016–2017

**DOI:** 10.15585/mmwr.mm6714a3

**Published:** 2018-04-13

**Authors:** Tracie J. Gardner, Ousmane M. Diop, Jaume Jorba, Smita Chavan, Jamal Ahmed, Abhijeet Anand

**Affiliations:** ^1^Polio Eradication Department, World Health Organization, Geneva, Switzerland; ^2^Global Immunization Division, CDC; ^3^Division of Viral Diseases, CDC.

Global efforts to eradicate polio began in 1988, and four of the six World Health Organization (WHO) regions currently have achieved poliofree certification. Within the remaining two regions with endemic poliomyelitis (African and Eastern Mediterranean), Afghanistan, Nigeria, and Pakistan have never interrupted transmission of wild poliovirus (WPV). The primary means of detecting poliovirus transmission is surveillance for acute flaccid paralysis (AFP) among children aged <15 years, combined with collection and testing of stool specimens for detection of WPV and vaccine-derived polioviruses (VDPVs)* in WHO-accredited laboratories within the Global Polio Laboratory Network (GPLN) (*1*,*2*). AFP surveillance is supplemented by environmental surveillance for polioviruses in sewage from selected locations. Genomic sequencing of isolated polioviruses enables the mapping of transmission by time and place, assessment of potential gaps in surveillance, and identification of the emergence of VDPVs (*3*). This report presents poliovirus surveillance data from 2016–2017, with particular focus on six countries in the Eastern Mediterranean Region (EMR) and 20 countries in the African Region (AFR) that reported WPV or circulating VDPVs (cVDPVs) during 2011–2017. Included in the 20 AFR countries are the three most affected by the 2014–2015 Ebola virus disease (Ebola) outbreak (Guinea, Liberia, and Sierra Leone), even though only one (Guinea) reported WPV or cVDPVs during the surveillance period. During 2017, a total of 14 (70%) of the 20 AFR countries and five (83%) of the six EMR countries met both surveillance quality indicators at the national level; however, provincial-level variation was seen. Surveillance strengthening activities are needed in specific countries of these regions to provide evidence supporting ultimate certification of the interruption of poliovirus circulation.

## Acute Flaccid Paralysis Surveillance

Two principal indicators measure the quality of AFP surveillance. The first is the nonpolio AFP (NPAFP) rate (i.e., the number of NPAFP cases per 100,000 children aged <15 years per year); an NPAFP rate ≥2 is considered sufficiently sensitive to detect WPV or VDPV cases if poliovirus is circulating. The second indicator is the collection of adequate stool specimens from ≥80% of patients with AFP (*2*). Adequacy refers to collection of two stool specimens ≥24 hours apart, within 14 days of paralysis onset, and arrival at a WHO-accredited laboratory in good condition.^†^

Among all 47 AFR countries evaluated, 31,759 AFP cases were reported in 2016 and 30,889 in 2017. No WPV type 1 (WPV1) cases were reported in AFR in 2017. The four WPV1 cases that occurred in AFR in 2016 were reported from Borno state in Nigeria (*4*). Although no AFP cases or environmental isolates of WPV1 have been detected in Borno for >1 year, it is difficult to determine if transmission of WPV1 persists in pockets of the population where polio surveillance is infeasible or limited (e.g., in insurgent-controlled and inaccessible areas) (*5*). One cVDPV case was reported in AFR during 2016, a cVDPV type 2 (cVDPV2) case from Nigeria. During 2017, a total of 22 cVDPV cases were reported in AFR, all cVDPV2 cases from the Democratic Republic of the Congo (Table 1). Among the 20 countries evaluated in AFR, 14 (70%) met both national surveillance indicators in 2017, compared with 12 (60%) in 2016. All three Ebola-affected countries had NPAFP rates ≥2 during 2016 and 2017. In 2016 only Guinea also achieved ≥80% stool adequacy; however in 2017, Guinea and Liberia both achieved ≥80% stool adequacy. 

**TABLE 1:** National and subnational acute flaccid paralysis surveillance indicators and number of confirmed wild poliovirus and circulating vaccine-derived poliovirus cases, by country, for all countries with poliovirus transmission during 2011–2017 and those that were affected by the Ebola virus disease outbreak in West Africa — World Health Organization African Region and Eastern Mediterranean Region, 2016–2017*

WHO Region/Country	No. of AFP cases (all ages)	Regional/National NPAFP rate^†^	% Subnational areas with NPAFP rate ≥2^§^	% Regional or national AFP cases with adequate specimens^¶^	% Subnational areas with ≥80% adequate specimens	% Population living in areas meeting both indicators**	No. of confirmed WPV cases*	No. of confirmed cVDPV cases*^,††^
**2016**								
**AFR (all 47 countries)^§§^**	31,759	7.4	NA	92	NA	NA	4	1
Angola	392	3.5	94	94	100	84	—^¶¶^	—^¶¶^
Cameroon	868	7.8	100	87	90	82	—	—
Central African Republic***	143	7.0	100	73	29	25	—	—
Chad	484	7.2	87	85	65	78	—	—
Côte d'Ivoire	371	4.2	85	94	85	74	—	—
DRC***	1,819	5.1	100	78	50	56	—	—
Equatorial Guinea	3	0.6	0	0	0	0	—	—
Ethiopia***	1,048	2.5	82	79	46	9	—	—
Gabon***	43	6.1	100	26	10	3	—	—
Guinea	1,061	20.1	100	88	88	85	—	—
Kenya	554	2.8	89	89	79	70	—	—
Liberia	69	3.6	100	75	53	43	—	—
Madagascar	791	7.6	96	86	77	80	—	—
Mali	307	3.8	89	90	78	96	—	—
Mozambique	425	3.2	90	82	40	59	—	—
Niger***	366	3.5	75	62	13	3	—	—
Nigeria	17,867	20.7	97	99	97	99	4	1
Republic of the Congo	82	3.6	83	82	67	78	—	—
Sierra Leone	68	2.6	100	77	50	45	—	—
South Sudan	323	6.3	90	91	80	70	—	—
**EMR (all 21 countries)^†††^**	15,951	7.6	NA	90	NA	NA	33	1
Afghanistan	2,905	20.1	100	92	97	100	13	—
Iraq	605	4.2	90	81	58	44	—	—
Pakistan	7,848	12.6	100	87	88	99	20	1
Somalia	316	5.9	100	99	100	100	—	—
Syria	246	3.2	57	81	64	33	—	—
Yemen	715	7.1	100	91	100	100	—	—
**2017**								
**AFR^§§^**	30,889	7.1	NA	92	NA	NA	—	22
Angola	411	3.6	94	97	100	84	—	—
Cameroon	973	8.9	100	85	90	82	—	—
Central African Republic	167	8.3	100	80	43	48	—	—
Chad***	702	10.2	100	79	52	62	—	—
Côte d'Ivoire	334	3.6	60	91	75	58	—	—
DRC***	2,113	5.8	100	79	46	42	—	22
Equatorial Guinea	12	3.7	57	17	14	0	—	—
Ethiopia	1,096	2.6	73	86	100	90	—	—
Gabon***	51	6.9	100	59	50	35	—	—
Guinea	453	8.4	100	87	100	100	—	—
Kenya	463	2.2	66	84	72	53	—	—
Liberia	81	4.1	100	82	60	76	—	—
Madagascar	701	6.6	100	93	96	99	—	—
Mali	256	3.1	100	88	89	95	—	—
Mozambique	374	2.9	100	86	70	80	—	—
Niger***	681	6.4	100	70	0	0	—	—
Nigeria	15,967	18.5	97	98	97	99	—	—
Republic of the Congo	118	5.5	83	84	58	66	—	—
Sierra Leone***	75	2.8	100	77	75	77	—	—
South Sudan	388	7.3	90	85	70	67	—	—
**EMR^†††^**	19,035	9.0	NA	88	NA	NA	22	74
Afghanistan	3,090	21.3	100	94	100	100	14	—
Iraq	699	4.8	95	87	79	74	—	—
Pakistan	10,196	16.3	100	86	100	100	8	—
Somalia	345	6.3	100	99	100	100	—	—
Syria***	348	3.6	57	70	57	28	—	74
Yemen	713	7.0	100	82	70	68	—	—

**Abbreviations:** AFP = acute flaccid paralysis; AFR = African Region; cVDPV = circulating vaccine-derived poliovirus; DRC = Democratic Republic of the Congo; Ebola = Ebola virus disease; EMR = Eastern Mediterranean Region; NA = not available; NPAFP = nonpolio AFP; WHO = World Health Organization; WPV = wild poliovirus.

* Data current as of February 22, 2018.

^†^ Per 100,000 persons aged <15 years per year.

^§^ For all subnational areas regardless of population size.

^¶^ Standard WHO target is adequate stool specimen collection from ≥80% of AFP cases, assessed by timeliness and condition. For this analysis, timeliness was defined as two specimens collected ≥24 hours apart (≥1 calendar day in this data set), and both within 14 days of paralysis onset. Good condition was defined as arrival of specimens in a WHO-accredited laboratory with reverse cold chain maintained and without leakage or desiccation.

** Percentage of the country’s population living in subnational areas which met both surveillance indicators (NPAFP rates ≥2 per 100,000 persons aged <15 years per year and ≥80% of AFP cases with adequate specimens).

^††^ cVDPV was associated at least one case of AFP with evidence of transmission and genetically linked. Guidelines for classification of cVDPV can be found at http://polioeradication.org/wp-content/uploads/2016/09/Reporting-and-Classification-of-VDPVs_Aug2016_EN.pdf.

^§§^ Algeria, Angola, Benin, Botswana, Burkina Faso, Burundi, Cameroon, Cabo Verde, Central African Republic, Chad, Comoros, Côte d’Ivoire, Democratic Republic of the Congo, Equatorial Guinea, Eritrea, Ethiopia, Gabon, Gambia, Ghana, Guinea, Guinea-Bissau, Kenya, Lesotho, Liberia, Madagascar, Malawi, Mali, Mauritania, Mauritius, Mozambique, Namibia, Niger, Nigeria, Rwanda, Republic of the Congo, Sao Tome and Principe, Senegal, Seychelles, Sierra Leone, South Africa, South Sudan, Swaziland, Togo, Uganda, Tanzania, Zambia, and Zimbabwe.

^¶¶^ Dashes indicate that no confirmed cases were found.

*** Stool adequacy dropped to <80% when stool condition was included with timeliness.

^†††^ Afghanistan, Bahrain, Djibouti, Egypt, Iran, Iraq, Jordan, Kuwait, Lebanon, Libya, Morocco, Oman, Pakistan, Qatar, Saudi Arabia, Somalia, Sudan, Syria, Tunisia, United Arab Emirates, and Yemen.

Among the 21 EMR countries, 15,951 AFP cases were reported in 2016, and 19,035 in 2017. Two EMR countries (Afghanistan and Pakistan) reported WPV1 cases in 2016 (33) and 2017 (22). The number of WPV1 cases reported by Afghanistan remained constant (13 in 2016 and 14 in 2017); the number reported from Pakistan declined from 20 (2016) to eight (2017). In 2016, one cVDPV2 case was reported in EMR, in Pakistan. In contrast, during 2017, 74 cVDPV cases were reported from EMR. All cases were type 2 and occurred in Syria; the most recent case occurred in September 2017 (Table 1), resulting in the largest cVDPV2 outbreak since the synchronized global cessation of use of type 2 oral poliovirus vaccine in April 2016 (*6*). Among the six countries evaluated in EMR, five met both national surveillance indicators in 2017, compared with all six in 2016 (Table 1). Although overall performance improved in 2017, national-level surveillance indicators masked suboptimal surveillance performance at subnational levels in both regions, (Table 1) (Figure).

**FIGURE:**
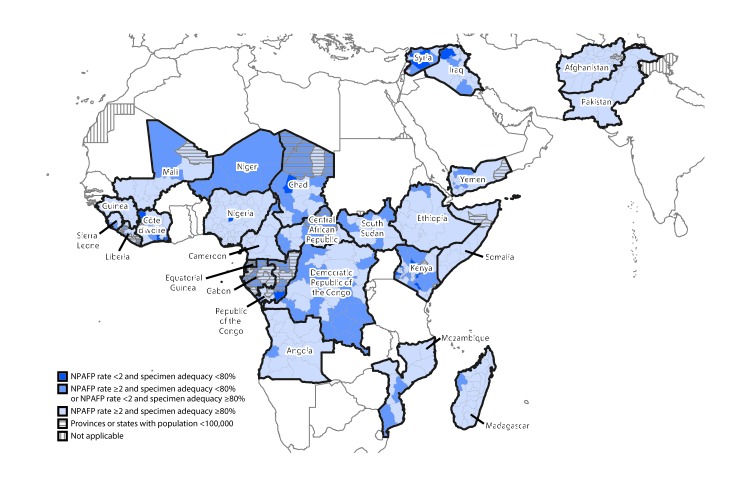
Combined performance indicators for the quality of acute flaccid paralysis surveillance in subnational areas (states and provinces) of 26 countries that had poliovirus transmission during 2011–2017 or were affected by the Ebola outbreak in West Africa during 2014–2015 — World Health Organization African and Eastern Mediterranean Regions, 2017*^,†^ **Abbreviations: **AFP = acute flaccid paralysis; NPAFP = nonpolio AFP. * The Global Polio Eradication Initiative has set the following targets for countries with current or recent wild poliovirus transmission and their states/provinces: 1) NPAFP detection rate of ≥2 cases per 100,000 persons aged <15 years per year and 2) adequate stool specimen collection from ≥80% of AFP cases, with specimen adequacy assessed by timeliness and condition. Timeliness was defined as two specimens collected ≥24 hours apart (≥1 calendar day) and both within 14 days of paralysis onset. Good condition was defined as specimens arriving without leakage or desiccation in a maintained reverse cold chain at a World Health Organization-accredited laboratory. ^†^ Data are for AFP cases with onset during 2017, reported as of February 22, 2018.

## Environmental Surveillance

Sewage sample testing supplements AFP surveillance by identifying poliovirus transmission that might occur in the absence of detected AFP cases (*3*,*6*). Environmental surveillance collection sites increased in Afghanistan, Nigeria, and Pakistan, from 21 in 2011 to 143 in 2017. As part of the Global Polio Eradication Initiative’s global environmental surveillance expansion plan, environmental surveillance is conducted in 91 sites in 38 countries without recent active WPV transmission, including 16 countries on the African continent.

In Nigeria, sewage sampling is currently conducted at 70 sites in 18 states and the Federal Capital Territory. No WPV has been isolated since May 2014, and cVDPV2 was last detected in Borno state in March 2016. In Afghanistan, environmental sampling is conducted at 20 sites in nine provinces; five of the 20 sites were added in 2017. WPV1 from four genetic clusters was detected in samples collected among five provinces in 2017. In Pakistan, sampling is conducted at 53 sites in five provinces, including the Islamabad Capital Territory; two of the 53 sites were added in 2017. In 2017, 13% of samples were positive for WPV1, compared with 11% in 2016. WPV1 was detected in all five provinces in 2017. Environmental sampling was established in Mogadishu, Somalia, in October 2017, and two of the first three samples collected yielded cVDPV2 isolates.

## Global Polio Laboratory Network

GPLN consists of 146 poliovirus laboratories located in the six WHO regions that are subject to a WHO-led quality assurance program. GPLN member laboratories follow standardized protocols to 1) isolate and identify poliovirus, 2) conduct intratypic differentiation (ITD) to identify WPV or screen for Sabin (vaccine) poliovirus and VDPV (*7*), and 3) conduct genomic sequencing. Sequencing results help monitor pathways of poliovirus transmission by comparing the nucleotide sequence of the VP1-coding region of poliovirus isolates. To meet standard laboratory timeliness indicators for processing a stool specimen, laboratories should report ≥80% of poliovirus isolation results within 14 days of specimen receipt, ≥80% of ITD results within 7 days of isolate receipt, and ≥80% of sequencing results within 7 days of ITD result. The standard combined field and laboratory performance indicator is to report ITD results for ≥80% of isolates within 60 days of paralysis onset in AFP cases. This indicator considers the entire interval from paralysis onset to specimen testing (EMR countries use a 45-day timeliness standard). The accuracy and quality of testing at GPLN laboratories is monitored through an annual accreditation program of onsite reviews and proficiency testing. During 2017, an accreditation checklist, including standard laboratory timeliness indicators for sewage sample processing, was implemented for laboratories conducting environmental surveillance.

GPLN tested 218,478 stool specimens from patients with AFP in 2016 and 201,546 in 2017. WPV1 was isolated from 37 AFP case samples in 2016 and 22 AFP case samples in 2017. In addition, cVDPV was detected from 11 AFP cases in 2016 and 96 in 2017. GPLN laboratories met timeliness indicators for poliovirus isolation in all regions (Table 2). The overall timeliness indicator for onset to ITD results was met in all regions in both years.

**TABLE 2:** Number of poliovirus isolates from stool specimens of persons with acute flaccid paralysis and timeliness of virus isolation and intratypic differentiation* reporting, by World Health Organization region — worldwide, 2016–2017^†^

WHO region/Year	No. of specimens	No. of poliovirus isolates			% Poliovirus isolation results on time**	% ITD results within 7 days of laboratory receipt^††^	% ITD results within 60 days of paralysis onset
		Wild	Sabin^§^	cVDPV^¶^			
**African**							
2016	65,520	4	4,771	4	95	94	97
2017	65,245	0	1,663	22	97	80	98
**Americas**							
2016	1,920	0	18	0	84	92	91
2017	1,755	0	14	0	83	100	100
**Eastern Mediterranean**							
2016	31,928	33	1,612	1	94	98	98
2017	35,602	22	2,521	74	98	99	97
**European**							
2016	3,606	0	71	0	82	100	86
2017	3,480	0	73	0	83	92	90
**South-East Asia**							
2016	101,550	0	5,247	2	98	99	99
2017	82,292	0	2,251	0	91	96	99
**Western Pacific**							
2016	14,196	0	253	4	96	98	96
2017	13,370	0	140	0	96	97	90
**Total^§§^**							
**2016**	**218,478**	**37**	**11,972**	**11**	**96**	**97**	**98**
**2017**	**201,546**	**22**	**6,662**	**96**	**94**	**91**	**98**

**Abbreviations:** cVDPV = circulating vaccine-derived poliovirus; ITD = intratypic differentiation; PV = poliovirus; PV1 = PV type 1; PV2 = PV type 2; VDPV = vaccine-derived poliovirus; WHO = World Health Organization.

* ITD is used to identify Sabin (vaccine) and non–Sabin-like poliovirus and screen for VDPV.

^†^ Data current as of February 28, 2018.

^§^ Either 1) concordant Sabin-like results in ITD test and VDPV screening or 2) ≤1% VP1 nucleotide sequence difference compared with Sabin vaccine virus (≤0.6% for VP2).

^¶^ For poliovirus types 1 and 3, ≥10 VP1 nucleotide differences from the respective poliovirus; for poliovirus type 2, ≥6 VP1 nucleotide differences from Sabin PV2.

** Results reported within 14 days of receipt of specimen.

^††^ Results of ITD reported within 7 days of receipt of specimen.

^§§^ For the last three indicators, total represents weighted mean percentage of regional performance.

Overall genetic diversity declined among WPV1 isolates in 2017. In 2017, South Asia (SOAS) genotype was the only WPV1 genotype circulating globally and was detected in Afghanistan and Pakistan. West Africa B1 (WEAF-B1) genotype was last detected in Nigeria in 2016. Sequence analysis associated with the SOAS genotype indicates that WPV1 cases might have been missed by AFP surveillance in 2017; orphan WPV1 isolates (those with less genetic relatedness [≤98.5% in VP1 gene] to other circulating viruses) were associated with three of 22 WPV1 cases reported from Afghanistan and Pakistan, indicating possible gaps in AFP surveillance. In 2017, cVDPV viruses with extended divergence from the parental Sabin strain were also isolated from stool specimens of AFP cases and from environmental samples in three countries.

## Discussion

The number of reported WPV cases declined to the lowest point ever in 2017; however, reported cVDPV cases increased from 2016 to 2017 because of major cVDPV2 outbreaks in the Democratic Republic of the Congo and Syria. Although most national-level surveillance quality indicators improved in 2017, considerable variation exists at subnational levels, particularly in inaccessible areas, and timely detection of circulating polioviruses can be hampered if active surveillance efforts are not rigorous. Repeated detection of WPV and cVDPV from sewage samples in locations where poliovirus cases have not been detected or where sewage detections have preceded detection in persons can provide early evidence of viral circulation within a community (e.g., WPV isolation in Pakistan during 2017) (*8*). Strategies to strengthen AFP surveillance in areas where conflict occurs have included increased AFP case searches among camps for internally displaced persons, engagement of community members in inaccessible areas, and active case searches in newly accessible areas (*5*). Although conflict might limit access to standard health facility–based surveillance, community-based surveillance has been effective in finding AFP cases, providing some assurance of the absence of poliovirus circulation in critical areas. For example, in Somalia, community volunteers have been instrumental in reporting AFP cases in inaccessible and partially accessible areas (*9*).

The findings in this report are subject to at least two limitations. First, security-related issues, issues associated with mobile and difficult-to-access populations, or other factors that affect surveillance performance could affect interpretation of AFP surveillance indicators. Second, high NPAFP rates do not necessarily imply sensitive surveillance, because a proportion of reported AFP cases might not be actual AFP cases, and not all actual AFP cases might be detected.

Certification of poliofree status requires at least 3 years of timely and sensitive poliovirus surveillance (*10*), including timely stool specimen collection and timely and appropriate transport of specimens to the laboratory. In 2017, specimen condition was a concern in Chad, DRC, Gabon, Niger, Sierra Leone, and Syria. Use of mobile technologies to improve timeliness and accuracy of AFP reporting in geographically hard-to-reach areas might be useful in some countries when linked with vigorous specimen collection (*5*). Strong supervision and monitoring of surveillance performance, especially at subnational levels, is important to achieve high-quality surveillance that can detect poliovirus transmission. Environmental surveillance has been an important supplement to AFP surveillance and, when carefully conducted in populations covered by sewage networks, can improve detection of circulating virus, particularly in high-risk areas with suboptimal AFP surveillance (*3*). Polio surveillance efforts need to reach geographically difficult-to-access and security-compromised areas and mobile and migrant populations. Surveillance data should be assessed routinely to identify suboptimal data quality. The need for strong poliovirus surveillance will continue beyond certification of eradication, until well after the use of all oral poliovirus vaccine has stopped globally. Poliovirus surveillance will need to be integrated with surveillance of other vaccine-preventable diseases to sustain capacity and maintain sufficient performance quality. As long as polioviruses continue to circulate in any country, all countries remain at risk.

**Summary**

**What is already known about this topic?**

Surveillance is the cornerstone of polio eradication efforts.

**What is added by this report?**

In 2017, 22 wild poliovirus cases were reported from two countries (Afghanistan and Pakistan), the fewest number ever reported globally. Polio cases caused by circulating vaccine-derived polioviruses increased from four in 2016 to 96 in 2017 because of large outbreaks in Syria and the Democratic Republic of the Congo. Although surveillance performance indicators are improving at the national level, gaps remain, including at subnational levels.

**What are the implications for public health practice?**

As polio cases decline, sensitive and timely surveillance becomes even more important. As long as polioviruses circulate in any country, all countries remain at risk.

## Footnotes

* Viruses that differ genetically from vaccine viruses and can emerge in areas with low vaccination coverage and cause paralysis.

† Reverse cold chain maintained and received without leakage or desiccation at a WHO-accredited laboratory. Reverse cold chain is maintained when stool specimens are stored immediately after collection at 4–8°C (32–39°F), frozen at -20°C (-4°F) when received for processing, and shipped to a WHO-accredited laboratory in dry ice or cold packs. Freezing of specimens is unnecessary if specimens can be received at a WHO-accredited laboratory within 72 hours of collection.

## References

[1] Maes EF, Diop OM, Jorba J, Chavan S, Tangermann RH, Wassilak SG. Surveillance systems to track progress toward global polio eradication—worldwide, 2012–2013. MMWR Morb Mortal Wkly Rep 2017;66:359–65. 10.15585/mmwr.mm6613a3 28384129 PMC5657908

[2] World Health Organization. WHO-recommended surveillance standard of poliomyelitis. Geneva, Switzerland: World Health Organization; 2015. http://www.who.int/immunization/monitoring_surveillance/burden/vpd/surveillance_type/active/poliomyelitis_standards/en/

[3] Asghar H, Diop OM, Weldegebriel G, Environmental surveillance for polioviruses in the Global Polio Eradication Initiative. J Infect Dis 2014;210(Suppl 1):S294–303. 10.1093/infdis/jiu384 25316848 PMC10578309

[4] Nnadi C, Damisa E, Esapa L, Continued endemic wild poliovirus transmission in security-compromised areas—Nigeria, 2016. MMWR Morb Mortal Wkly Rep 2017;66:190–3. 10.15585/mmwr.mm6607a2 28233765 PMC5657850

[5] Bolu O, Nnadi C, Damisa E, Progress toward poliomyelitis eradication—Nigeria, January–December 2017. MMWR Morb Mortal Wkly Rep 2018;67:253–6. 10.15585/mmwr.mm6708a5 29494568 PMC5861699

[6] Hampton LM, Farrell M, Ramirez-Gonzalez A, ; Immunization Systems Management Group of the Global Polio Eradication Initiative. Cessation of trivalent oral poliovirus vaccine and introduction of inactivated poliovirus vaccine worldwide, 2016. MMWR Morb Mortal Wkly Rep 2016;65:934–8. 10.15585/mmwr.mm6535a3 27606675

[7] Kilpatrick DR, Yang CF, Ching K, Rapid group-, serotype-, and vaccine strain-specific identification of poliovirus isolates by real-time reverse transcription-PCR using degenerate primers and probes containing deoxyinosine residues. J Clin Microbiol 2009;47:1939–41. 10.1128/JCM.00702-09 19386844 PMC2691077

[8] Elhamidi Y, Mahamud A, Safdar M, Progress toward poliomyelitis eradication—Pakistan, January 2016–September 2017. MMWR Morb Mortal Wkly Rep 2017;66:1276–80. 10.15585/mmwr.mm6646a4 29166363 PMC5769788

[9] Polio Global Eradication Initiative. Poliovirus risk analysis for conflict-affected polio-free countries. EMRO—December 2016. Geneva, Switzerland: Polio Global Eradication Initiative; 2017. http://polioeradication.org/wp-content/uploads/2017/04/Risk-Assessment_Specific_EMR_Countries_2017.pdf

[10] World Health Organization. Report of the 1st meeting of the Global Commission for the Certification of the Eradication of Poliomyelitis. Geneva, Switzerland: World Health Organization; 1995. http://polioeradication.org/wp-content/uploads/2016/07/10Report.pdf

